# Downregulation of Fat Mass and Obesity Associated (FTO) Promotes the Progression of Intrahepatic Cholangiocarcinoma

**DOI:** 10.3389/fonc.2019.00369

**Published:** 2019-05-09

**Authors:** Zhuo-Xian Rong, Zhi Li, Jun-Ju He, Li-Yu Liu, Xin-Xin Ren, Jie Gao, Yun Mu, Yi-Di Guan, Yu-Mei Duan, Xiu-Ping Zhang, De-Xiang Zhang, Nan Li, Yue-Zhen Deng, Lun-Quan Sun

**Affiliations:** ^1^Center for Molecular Medicine, Xiangya Hospital, Central South University, Changsha, China; ^2^Key Laboratory of Molecular Radiation Oncology, Changsha, China; ^3^Hunan International Collaboration Base for Science and Technology, Changsha, China; ^4^Department of Pathology, Xiangya Hospital, Central South University, Changsha, China; ^5^Department of Hepatic Surgery VI (Ward I), Shanghai Eastern Hepatobiliary Surgery Hospital, Shanghai, China; ^6^General Surgery Department, Zhongshan-Xuhui Hospital Affiliated to Fudan University, Shanghai, China

**Keywords:** FTO, ICC, RNA m^6^A modification, metastasis, TEAD2

## Abstract

Intrahepatic cholangiocarcinoma (ICC) ranks as the second most malignant type of primary liver cancer with a high degree of incidence and a very poor prognosis. Fat mass and obesity-associated protein (FTO) functions as an eraser of the RNA m^6^A modification, but its roles in ICC tumorigenesis and development remain unknown. We showed here that the protein level of FTO was downregulated in clinical ICC samples and cell lines and that FTO expression was inversely correlated with the expression of CA19-9 and micro-vessel density (MVD). A Kaplan-Meier survival analysis showed that a low expression of *FTO* predicted poor prognosis in ICC. *in vitro*, decreased endogenous expression of *FTO* obviously reduced apoptosis of ICC cells. Moreover, *FTO* suppressed the anchorage-independent growth and mobility of ICC cells. Through mining the database, FTO was found to regulate the integrin signaling pathway, inflammation signaling pathway, epidermal growth factor receptor (EGFR) signaling pathway, angiogenesis, and the pyrimidine metabolism pathway. RNA decay assay showed that oncogene *TEAD2* mRNA stability was impaired by FTO. In addition, the overexpression of FTO suppressed tumor growth *in vivo*. In conclusion, our study demonstrated the critical roles of FTO in ICC.

## Introduction

Intrahepatic cholangiocarcinoma (ICC) is the second most malignant type of primary liver cancer, and its incidence is increasing (1, 2). Intrahepatic cholangiocarcinoma (ICC) originates from epithelial cells of the bile duct or hepatic ducts (3). Since some aspects of the clinical features are similar to those of hepatocellular carcinoma (HCC), ICC has insidious symptoms, a high degree of malignancy and very poor prognosis (4). ICC harbors many genetic aberrations, including mutations in isocitrate dehydrogenase1/2 (IDH1/2), epidermal growth factor receptor (EGFR), fibroblast growth factor receptor (FGFR), KRAS, and BRAF (5, 6), as well as amplifications of Cyclin D1 (*CCND1*). Strategies for ICC treatment include surgery, intervention, ablation treatment, radiotherapy, chemotherapy, immune targeting, and other comprehensive treatments (7–9). In principle, ICC is insensitive to radiotherapy and chemotherapy, and thus, the clinical outcomes are very poor.

N^6^-methyladenosine (m^6^A) represents a predominant RNA modification that, not surprisingly, regulates tumorigenesis, and tumor progression (10). The m^6^A RNA modification is a reversible process that is coordinated by methyltransferase (m^6^A “writers”), m^6^A reader proteins and demethylase (m^6^A “erasers”) (11). These members cover more than 13 enzymes. The m^6^A “writers” complex consists of METTL3, METTL14, WTAP, CBLL1, RBM15, ZC3H13, and VIRMA and is responsible for methylation of target RNA transcripts (12). Then, m^6^A readers, including YTHDF1-3, YTHDC1, IGF2BPs, and eIF3, discern these m^6^A modifications to direct RNA alternative splicing, translation, localization, and RNA stability, among other processes (13). However, as the m^6^A “erasers,” FTO and ALKBH5 remove m^6^A from the aforementioned target transcripts (14, 15). FTO, the first identified m^6^A demethylase, belongs to the AlkB family of Fe(II)/α-ketoglutarate-dependent dioxygenases (16). FTO mediates multiple RNA modifications, including m^6^A and m^6^A_m_ in mRNA and snRNA as well as m^1^A in tRNA (17). Recent studies demonstrated that FTO plays an oncogenic role in cancers (18–20). For example, in one study, FTO significantly promoted leukemic cell proliferation, transformation, leukemogenesis, curtailed AML cell differentiation and apoptosis by targeting ASB2, and RARA (18). However, this process may be suppressed by R-2-hydroxyglutarate (R-2HG) and IDH mutations, which competitively inhibit FTO (21, 22). FTO also removes m^6^A from β-catenin to induce the chemo-radiotherapy resistance in cervical squamous cell carcinoma (CSCC) (20). In addition, the overexpression of FTO can promote breast cancer and gastric cancer progression (19, 23). These studies suggest that FTO participates in the regulation of various biological processes in cancer cells through modulation of the RNA transcripts which are critical to the respective pathways.

In the present study, we sought to explore the clinical relevance and biological functions of FTO in ICC.

## Materials and Methods

### Cell Lines and Reagents

HIBEPIC, HCCC-9810, RBE, TFK-1, and HuCC-T1 cells (ATCC, Manassas, VA, USA) were cultured in RPMI-1640 medium supplemented with 10% fetal bovine serum. All cell lines were transfected with plasmids using *DharmaFECT Duo Transfection Reagent* (Thermo Scientific, Waltham, MA, USA) according to the manufacturer's instructions. Vectors expressing short hairpin RNA (shRNA) sequences were provided by Sangon Biotechnology (Shanghai, China). Cisplatin was obtained from Qilu Pharm (Jinan, China) was obtained from Hansoh (Jiangsu, China).

#### Western Blotting (WB)

For WB, 5 × 10^6^ cells were incubated with 100 μl cell lysis buffer (50 mM Tris-HCl (pH 8.0), 150 mM NaCl, 1% NP-40, 0.1% sodium deoxycholate, 0.1% SDS, and protease and phosphatase inhibitor cocktail (B14002&B15002, Biotool) at 4°C for 30 min. After centrifugation for 30 min, the supernatant was collected. All samples were mixed with 4 × SDS-PAGE loading buffer and analyzed by WB with the indicated antibody. The following primary antibodies were used in this study: Anti-FTO (1:4000, ab124892, Abcam), Anti-α-Tubulin (1:1000, sc-69969, Santa Cruz), and Anti-FLAG (1:5000, F1804, SIGMA).

#### Expression Plasmids and Clones

Human FTO was generated using the following primers: forward: 5′-ATGAAGCGCACCCCGACTGC-3′; reverse: 5′-CTAGGGTTTTGCTTCCAGAA-3′. siRNA sequence 1: 5′-TCACCAAGGAGACTGCTATTT-3′; siRNA sequence 2: 5′-CTAGGGTTTTGCTTCCAGAA-3′.

The vectors psPAX2 and pMD2.G (Addgene) were used for viral packaging. A small hairpin RNA (shRNA) expression plasmid pLVX-shRNA was used to construct lentivirus for the knockdown of FTO. The plasmids pLVX-IRES-Puro (Flag-SBP), pCDNA3.1^+^ and pRK5-FLAG were used for the overexpression of FTO.

#### Quantitative Reverse Transcriptase-PCR

RNA was extracted using TRIzol (Invitrogen). Then, a PrimeScript™ RT Reagent Kit with gDNA Eraser was used according to the manufacturer's protocol (TaKaRa). The primer sequences were as follows: FTO, forward primer: 5′-ACTTGGCTCCCTTATCTGACC-3′, reverse primer: 5′-TGTGCAGTGTGAGAAAGGCTT-3′. RT-PCR was performed using a CFX96 Real-Time PCR Detection System (Bio-Rad, Richmond, CA, USA). The 2^−ΔΔ^Ct method was used to calculate the expression of FTO. Three replicates were tested for each gene for each sample, and the mean value was calculated. The experiment was repeated three times.

#### Soft Agar Colony Forming Assay

Cells were seeded in 24-well culture plates (3 × 10^3^ cells/well, 4 repetitions per group) with Roswell Park Memorial Institute 1640 (RPMI-1640) containing 20% FBS. Agar was autoclaved and placed in a 42°C water bath kettle. Lower layer: 0.7% agar was mixed with 2 × RPMI-1640 (containing 2 × antibiotic and 20% FBS) at the ratio of 1:1, and 200 μl was added to each well of the 24-well plate, which was allowed to solidify in a CO_2_ incubator for at least 30 min. Upper layer: The 1.2% agar was also mixed with the cell suspension at a ratio of 1:1, and 200 μl was added to each well of the 24-well plate, which was maintained in a CO_2_ incubator. The soft agar colony plate was incubated in a 5% CO_2_ incubator at 37°C for 10~14 days. Eight fields were randomly selected for colony counts (>50 μm diameter) under an inverted microscope (Leica DMI4000B, Germany) at 40 × magnification. The freeware ImageJ (National Institutes of Health, Bethesda, MD, USA) and Adobe Photoshop (Adobe Systems, San Jose, CA, USA) were used in colony diameter measurement. Thirteen colonies were randomly selected for diameter measurement. Colony volume was calculated using the formula: volume = (length × width^2^)/2.

#### Flow Cytometry

For the apoptosis analysis, 5 × 10^5^ cells were digested with trypsin without EDTA and centrifuged (300 g, at 4°C for 5 min). Cells were washed twice in prechilled PBS. Then, 100 μl of 1 × Binding Buffer was added to resuspend the cells. Then, the cells were incubated with 5 μl Annexin V-FITC and 5 μl propidium iodide (PI) Staining Solution for 10 min at room temperature after gentle mixing. After adding 400 μl of 1 × Binding Buffer and mixing, the samples were examined for 1 h by flow cytometry. The apoptosis detection kit used in this experiment was purchased from BIomake (B32117, Houston, TX, USA).

#### Transwell Assay

The Transwell chamber (8 μm, Corning #3422, ME, USA) was placed into a 24-well plate. The transfected cells were diluted with 200 μl 0.1% FBS medium to generate a cell suspension with a density of 1 × 10^6^ cells/mL and seeded in the upper chamber. Then, 600 μl RPMI-1640 containing 30% FBS was added to the basolateral chamber. Each group was tested in 3 replicates. After culture at 37°C with 5% CO_2_ for 72 h, the Transwell chambers were removed, the cells were rinsed with PBS twice, fixed and stained with 0.3% crystal violet for 15 min, and rinsed in ddH_2_O three times. Cells in the upper chamber were removed. The invasive cells were observed with an inverted microscope (DMI 3000, Leica, IL, USA) (×100), and 5 fields were randomly selected for cell counts.

#### Immunohistochemistry (IHC)

Paraffin-embedded sections were deparaffinized and rehydrated. Antigen retrieval was performed by boiling the sections in sodium citrate solution in a pressure-cooker for 3 min. Endogenous peroxidase was blocked by 3% H_2_O_2_ solution for 10 min at 37°C and washed with PBST. Then, the paraffin sections were incubated with the primary antibody at 4°C overnight. The next day, the paraffin sections were rewarmed at 37°C for 10 min. After two washes in PBS and treatment with an adjuvant for 20 min at 37°C, the sections were incubated with the second antibody for 30 min at 37°C. The immunohistochemical reaction was visualized with 3,3,0-diaminobenzidine (DAB) for 3 min. Sections were counterstained for 3 min in hematoxylin (zSGB-Bio, Beijing). Slides were scanned with the digital 3DHISTECH–Pannoramic MIDI (3DHISTECH Ltd, Budapest). The image analysis software ImageJ IHC Profiler was used for staining quantification (24).

#### Colony Formation Assay

ICC cells were seeded in 12-well plates at densities of 2 × 10^2^ per well and. After 12 days culture, cells were treated with 0, 10 and 20 μM cisplatin. In 4 days later, cells were washed with cold phosphate buffered saline (PBS) and stained with 0.3% crystal violet (containing methanol). Colonies consisting of more than 50 cells were defined as surviving colonies. All viability measurements are normalized with the untreated group. Cell survival curves were fitted by GraphPad software.

#### RNA Decay Assay

FTO-overexpressing TFK1 cells, shFTO TFK1 cells, and corresponding control cells were cultured in 12-well plates. Then cells were treated with 8 μM actinomycin D (MedChemExpress, Monmouth Junction, NJ, USA) at 0, 2, and 4 h before RNA extraction.

#### Animal Assay

1.5 × 10^6^ TFK1 cells with or without FTO overexpression were injected subcutaneously into the left and right flanks of 6-week-old female athymic nude mice (SJA, Changsha, China). After transplantation, tumor size was measured by caliper every other day. Tumor volume was calculated using the formula: volume = (length × width^2^)/2. The animals were sacrificed and the tumors were removed for WB and IHC assay. All animal work procedures were performed in accordance with the guidelines of the Association for Assessment and Accreditation of Laboratory Animal Care International and approved by the Animal Ethics Committee of Xiangya Hospital, Central South University.

#### Statistical Analyses

In our study, Data is presented as mean ± SD, and analyzed using the two-tailed Student *t*-test for two groups, ANOVA for multiple groups or Chi-square tests for Table 1. The statistical significance of Kaplan–Meier survival curves was assessed with a Log-rank (Mantel-Cox) Test (25). A value of *p* < 0.05 was considered as statistically significant. ^*^P < 0.05, ^**^P < 0.01, ^***^P < 0.001. Student's *t*-tests, two-way ANOVA, curve fitting and box plots calculations were performed with GraphPad software (GraphPad, La Jolla, CA, USA). IBM SPSS Statistics 19 (IBM, Chicago, USA) is used for Chi-square test analysis (26).

**Table 1:** Relationship between FTO expression in ICC and clinicopathologic features.

**Variable**	**Cases**	**FTO expression**		***p*-value**
		**High**	**Low**	
Age
≤ 60	57	20	37	0.713
>60	80	55	25	
WBC(× 10^9^/L)
≤ 6.4	77	38	39	0.023
>6.4	60	42	18	
HGB(g/L)
≤ 160	35	12	23	< 0.001
>160	102	68	34	
RBC(× 10^12^/L)
≤ 4.48	49	33	16	< 0.001
>4.48	88	47	41	
AFU(μg/L)
≤ 17	50	33	17	0.01
>17	87	47	40	
CA19-9
≤ 40	70	48	22	0.016
>40	67	32	35	
ALP(U/L)
≤ 135	86	54	32	0.08
>135	51	26	25	
CD34
Positive	52	37	15	0.014
Negtive	85	43	42	

## Results

### FTO Protein Levels Are Decreased in ICC

**Figure 1:**
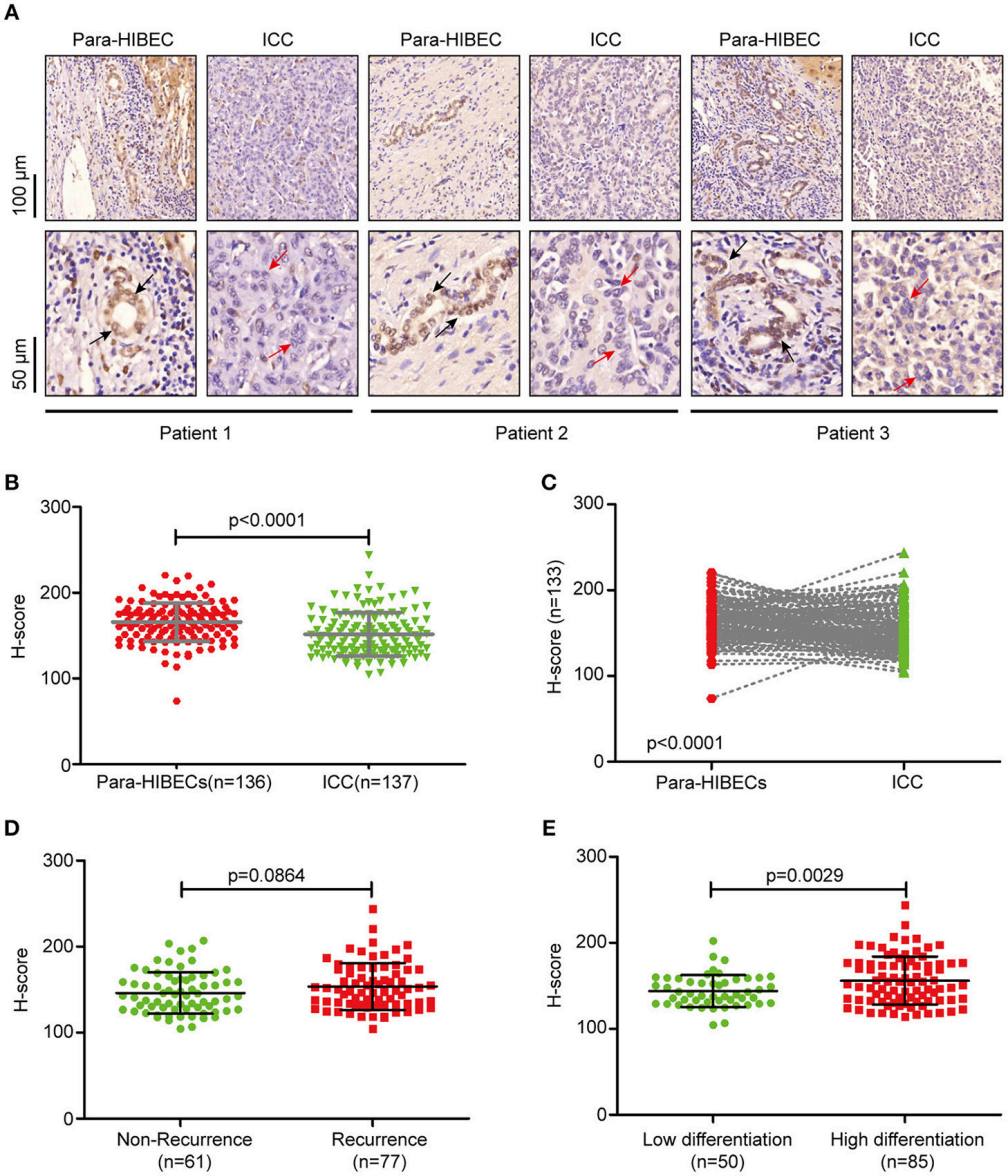
FTO was downregulated in intrahepatic cholangiocarcinoma. **(A)** Representative IHC staining of FTO in human ICC specimens. Black arrow indicates intrahepatic bile duct epithelial cells and red arrow indicates ICC cells. **(B)** Statistical analysis of FTO expression in ICC cases (*n* = 137) and human paracancerous intrahepatic biliary epithelial cells (para-HIBECS, *n* = 136). **(C)** Analysis of FTO expression between ICC cases and paired para-HIBECS (*n* = 133). **(D)** Comparison of FTO expression in the cases of recurrence (*n* = 77) with that of specimens from patients without recurrence (*n* = 6 1). **(E)** Differential expression of FTO between poorly-differentiated cases (*n* = 50) and highly differentiated cases (*n* = 85).

To understand the regulation of m^6^A modification in ICC, we examined the expression of m^6^A-related enzymes in human paracancerous intrahepatic biliary epithelial cells (para-HIBECS) and ICCs by IHC (data not shown). After analyzing the tissue microarray containing 142 patient specimens, it was found that FTO was downregulated in ICC (Figures 1A–C). Although no correlation was observed between disease recurrence and FTO expression (*p* = 0.084, Figure 1D), the expression of FTO was positively correlated with tumor differentiation, and a lower protein level was observed in the poorly differentiated ICC clinical samples (*p* = 0.0029, Figure 1E).

### FTO Expression Is Correlated With the Clinicopathologic Features of ICC

In the further analysis, we assessed the association between FTO expression and the clinicopathologic features. No association was observed between FTO expression and clinicopathologic features such as age, gender, cirrhosis, primary tumor diameter, tumor number, necrosis, and p53 or E-cadherin expression. Carbohydrate antigen 19-9 (CA19-9), a mucin-type glycoprotein in the serum, is one of the tumor markers used for ICC diagnosis, classification, prognosis, and treatment guidance (27, 28)^.^ A higher CA19-9 level in the serum is associated with poor prognosis. Our data indicated that the expression of FTO was negatively correlated with CA19-9 concentration (*p* = 0.016, Table 1). CD34, another tumor marker, is involved in angiogenesis and is used as a quantitative indicator of micro-vessel density (MVD) (29). An association was found between low FTO expression and high CD34 expression in ICC (*p* = 0.014, Table 1). In addition, FTO expression was also significantly related to the number of WBCs (white blood cells, *p* = 0.023) and RBCs (red blood cells, *p* < 0.001), HGB levels (hemoglobin, *p* < 0.001), AFU levels (α-L-Fucosidase, *p* = 0.01), and ALP level (alkaline phosphatase, *p* = 0.008) in the serum.

### Low Expression of FTO Predicts Poor Prognosis in ICC

**Figure 2:**
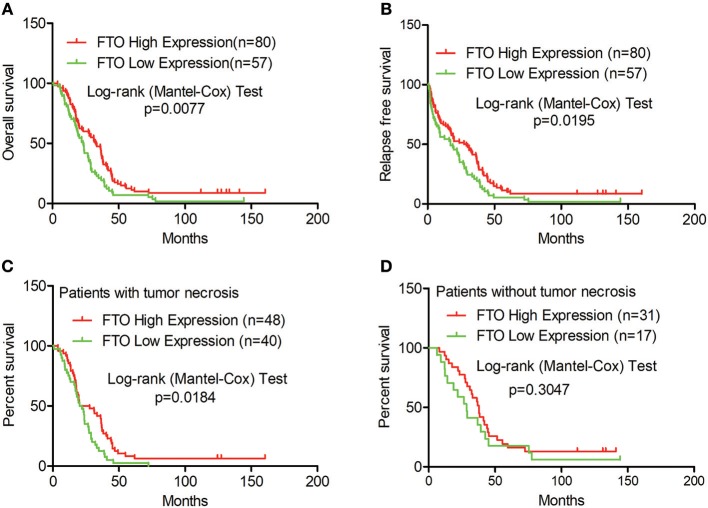
Low expression of FTO predicted a poor prognosis in ICC. **(A–D)** Kaplan-Meier survival analysis. Red line, FTO high expression; Green line, FTO low expression. **(A)** Overall survival analysis (*n* = 137), **(B)** Relapse-free survival analysis (*n* = 137), **(C)** Overall survival analysis in patients with tumor necrosis (*n* = 88) and **(D)** in tumor necrosis-free patients (*n* = 48).

Next, we analyzed the correlation between FTO expression and prognosis through the tissue microarray IHC staining described above. ICC patients with low expression of FTO showed both poorer overall survival (*p* = 0.0077) and poorer relapse-free survival (*p* = 0.0195) (Figures 2A,B). Moreover, in 88 patients with necrobiotic tumor, those with a lower expression of FTO also showed a poorer overall survival than those with a higher expression of FTO (*p* = 0.0273, Figure 2C). However, the overall survival of patients without necrosis did not differ between the lower FTO expression group and the higher FTO expression group (*p* = 0.1051, Figure 2D).

### FTO Promotes Cisplatin -Induced Apoptosis and Inhibits the Migration of ICC Cells *in vitro*

**Figure 3:**
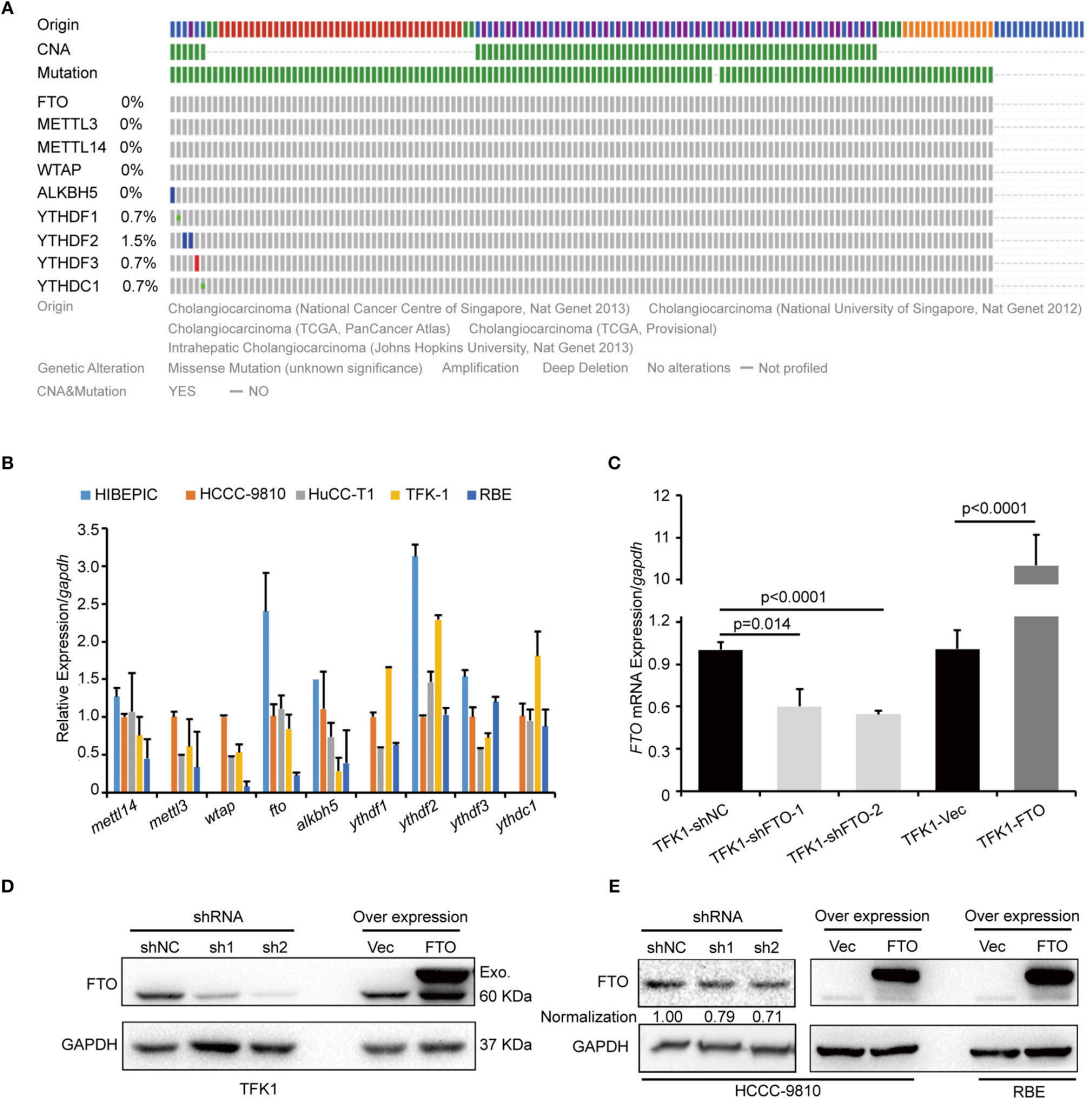
FTO mRNA level was low in ICC cells. **(A)** The genetic alterations of erasers, readers, and writers for the m^6^A modification in ICC were analyzed by cBioPortal. **(B)** The expression levels of erasers, readers and writers for m^6^A modification in ICC cell lines (*n* = 4) and the intrahepatic biliary epithelial cell line HIBEPIC were analyzed by q-PCR. **(C)** The mRNA levels of FTO in control, overexpression, and knockdown TFK1 cells were assessed by q-PCR. **(D,E)** The protein levels of FTO in control, overexpression and knockdown TFK1 and HCCC-9810 cells.

**Figure 4:**
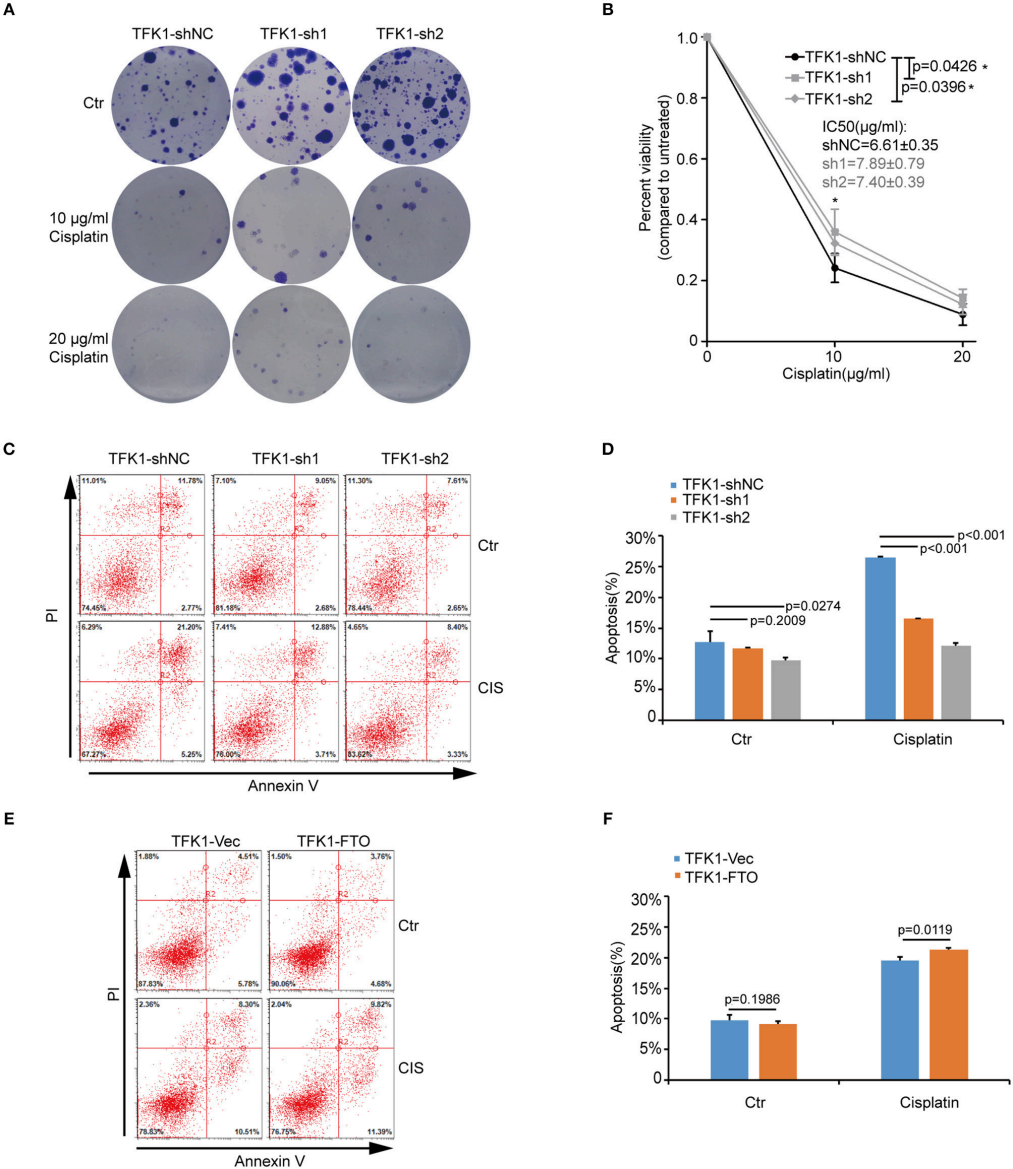
FTO promoted cisplatin-induced apoptosis of ICC cells. **(A,B)** Clonogenic survival assay of shFTO TFK1 cells and corresponding control cells treated with 0, 10, and 20 μM cisplatin. Data are presented as mean ± SD from *n* = 4. **(C)** The effects of FTO knockdown on the apoptosis of TFK1 cells treated with cisplatin (CIS, 30 ng/ml, 24 h) were examined using flow cytometric analysis. **(D)** The statistical analysis for **(C)**. Data are presented as mean ± SD from *n* = 3. **(E)** The effects of FTO over-expression on the apoptosis of TFK1 cells treated with cisplatin (CIS, 30 ng/ml, 24 h) were examined using flow cytometric analysis. **(F)** The statistical analysis for **(E)**, data are presented as mean ± SD from *n* = 3.

We next investigated the biological functions of *FTO* in ICC. We first analyzed *FTO* mutation status and copy number alterations (CNAs) in cholangiocarcinoma using the cBioPortal for Cancer Genomics (http://www.cbioportal.org). Neither mutations nor copy number alterations in *FTO* or other m^6^A modification enzymes were found in cholangiocarcinoma (Figure 3A). The mRNA level of *FTO* was lower in ICC cell lines (HCCC-9810, HuCC-T1, TFK1 and RBE) compared with normal human intrahepatic biliary epithelial cells (HIBEPIC, Figure 3B). TFK1 and HCCC-9810 cells were used for both gain- and loss-of-function assays (Figures 3C–E). Cisplatin, which is a standard chemotherapy regimen, has been extensively used for treating ICC and other cancers. To examine a potential function in chemosensitivity, we tested FTO knock-down cells in their response to cisplatin through assaying plating efficiency. Downregulating FTO insensitized the ICC cells to the cisplatin treatment (Figures 4A,B). Consistently, decreased endogenous expression of FTO obviously reduced apoptosis in ICC cell lines (Figures 4C,D), and increased expression of *FTO* synergized cisplatin-induced apoptosis in ICC cell lines (Figures 4E,F).

**Figure 5:**
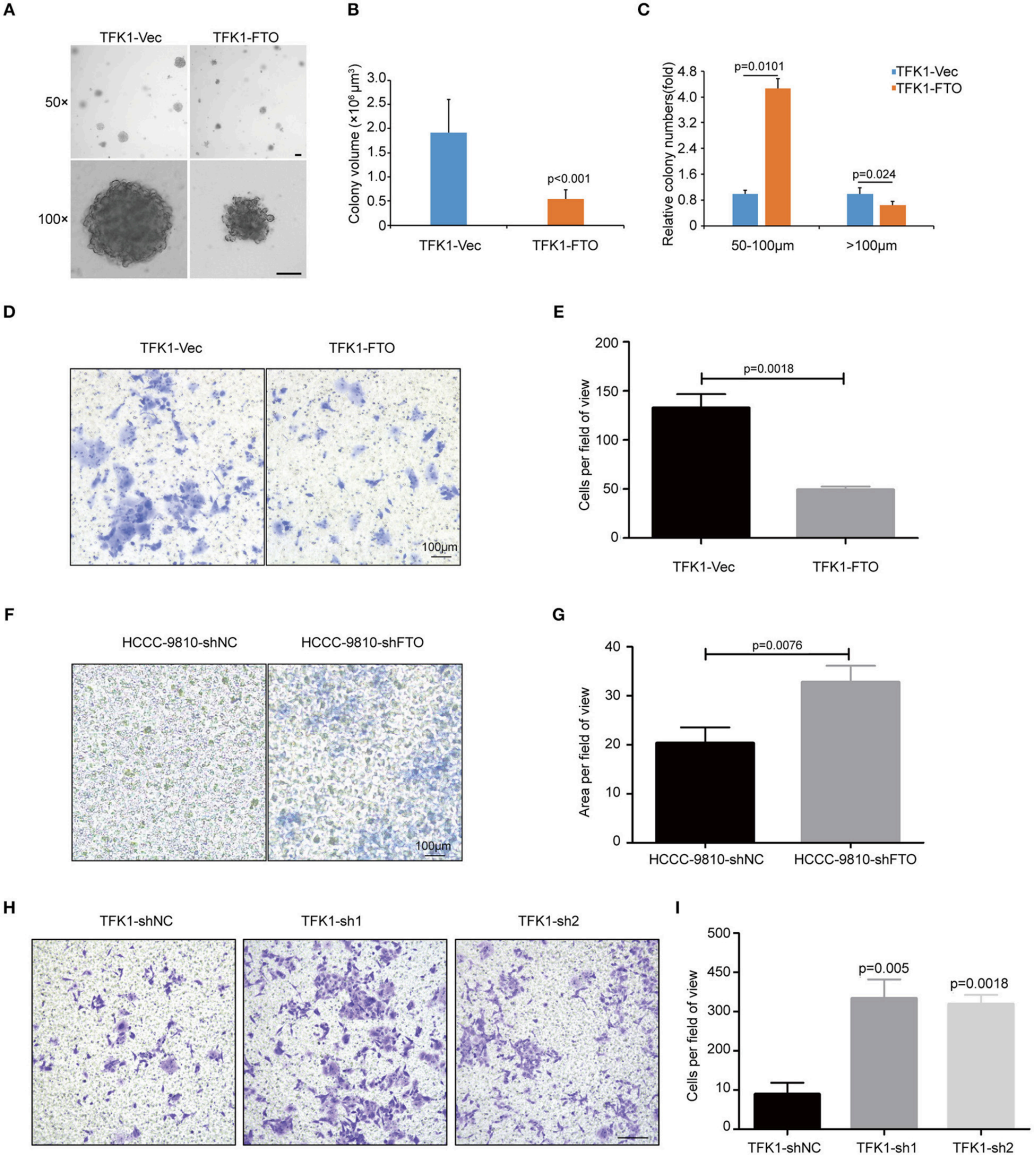
FTO inhibited the colony formation and migration of ICC cells *in vitro*. **(A)** The anchorage-independent viability of TFK1 cells was tested in a soft agar colony formation assay. **(B)** Statistical analysis of colony size. **(C)** Statistical analysis of colony numbers. **(D,E)** Transwell assays were performed to examine the effects of FTO overexpression on the invasiveness of FTK1 cells (scale: 100 μm). **(F–I)** Transwell assays were performed to examine the effects of FTO knockdown on the invasiveness of HCCC-9810 and TFK1 cells (scale: 100 μm).

To examine the effects of *FTO* expression on the anchorage-independent growth and invasion of ICC cells, we performed soft agar colony assay and Transwell migration assay. Forced expression of *FTO* dramatically reduced colony numbers and colony size compared with the control (Figures 5A–C). Additionally, ectopic expression of *FTO* significantly hampered the invasiveness of TFK1 cells (Figures 5D,E), and knockdown of FTO promoted the invasiveness of HCCC-9810 and TFK1 cells (Figures 5F–I).

### FTO Regulates ICC Progression Through Multiple Key Oncogenes and Suppressors

**Figure 6:**
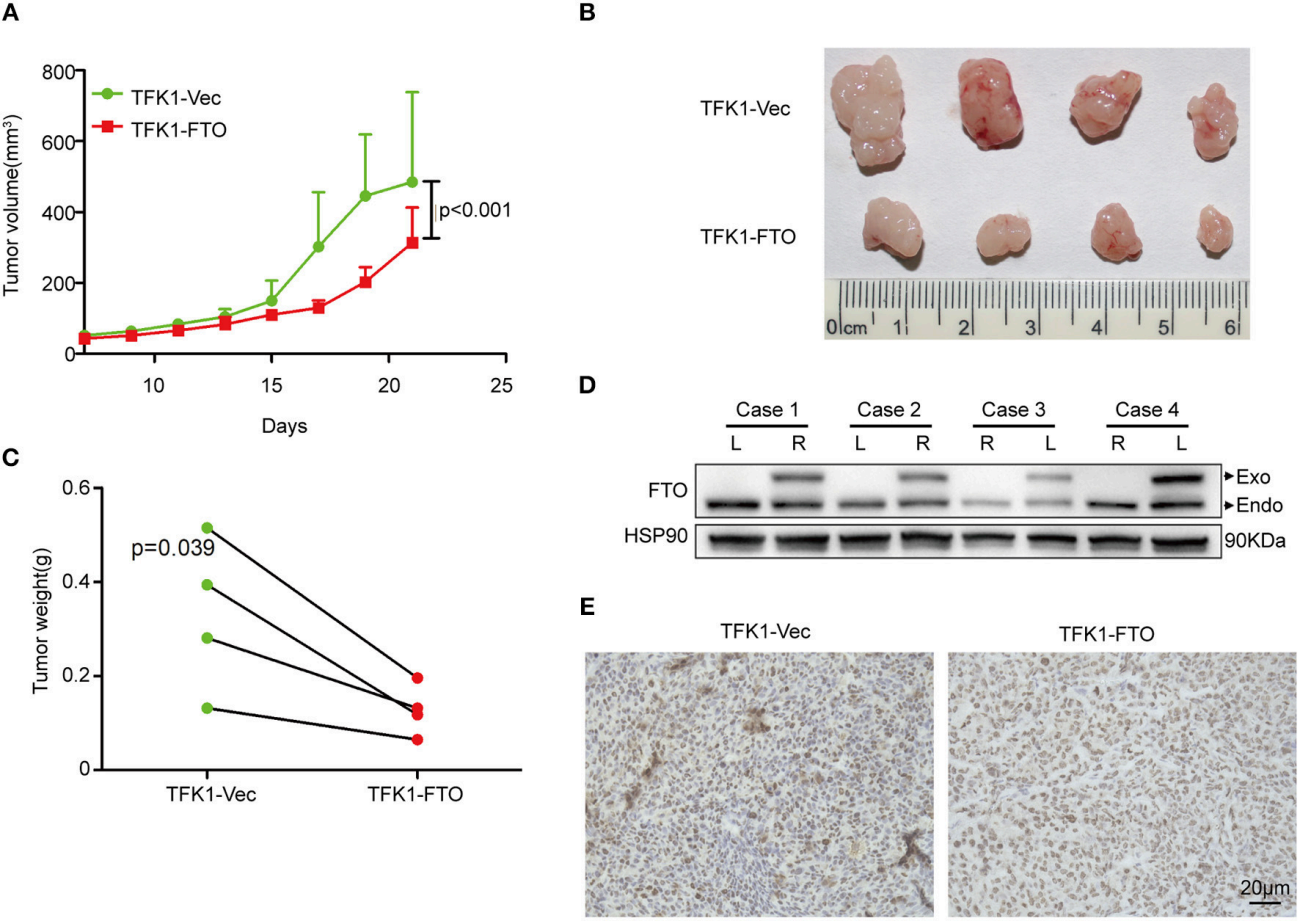
The overexpression of FTO suppressed tumor growth. **(A)** The growth curve of the tumor (*n* = 4). **(B)** FTO overexpression significantly suppressed tumor growth (*n* = 4). **(C)** Tumors weight (*n* = 4). **(D,E)** The FTO expression in tumors was determined by western blot **(D)** and immunohistochemistry **(E)**.

To determine the effects of FTO expression on ICC growth *in vivo*, TFK1 cells (control cells and FTO overexpressing cells) were subcutaneously transplanted into nude mice. Consistently, overexpression of FTO suppressed the growth, volume and weight of the tumors (Figures 6A–C). In addition, the overexpression of FTO in tumors was further determined by western blot and immunohistochemistry (Figures 6D,E).

**Figure 7:**
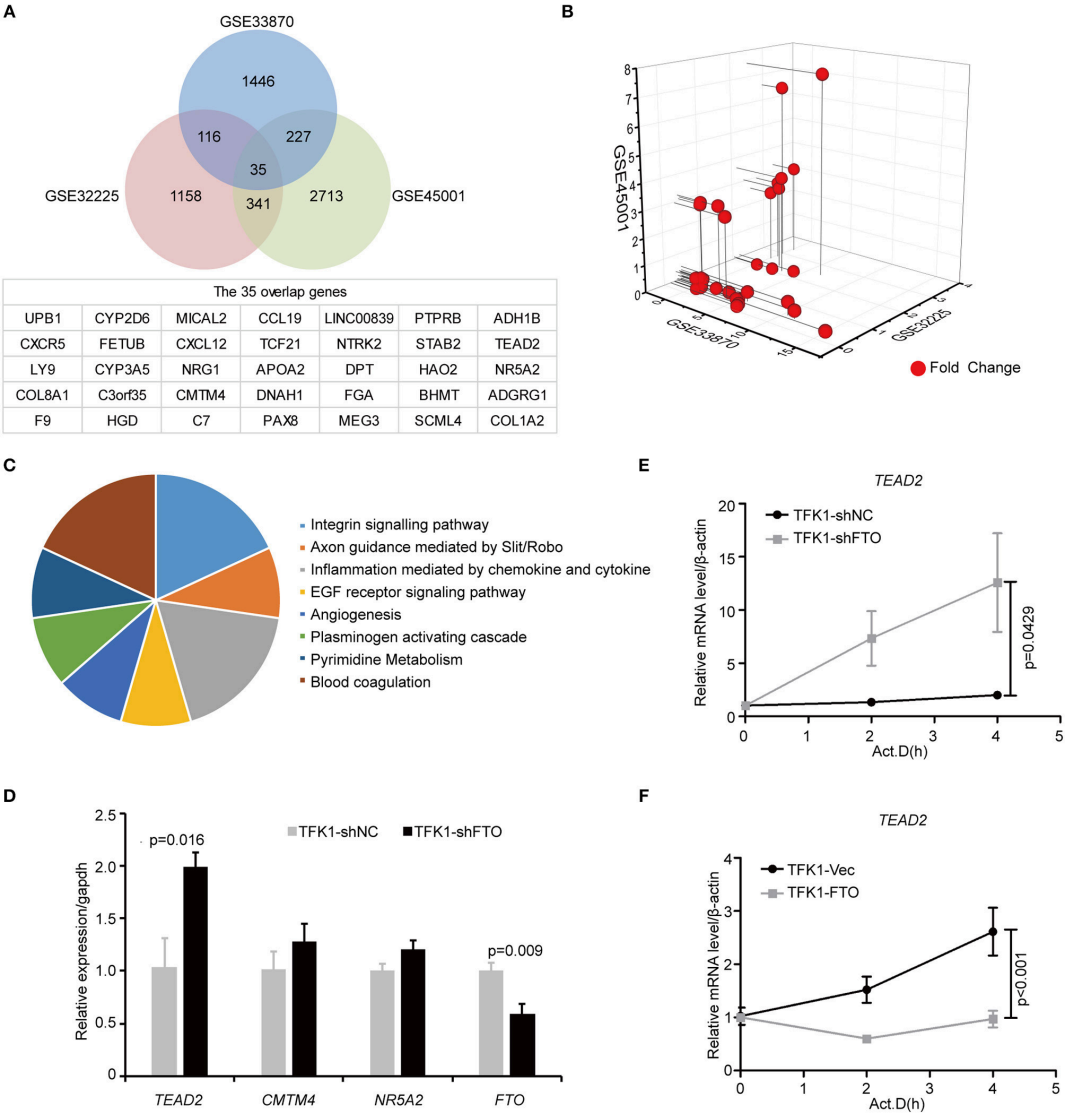
FTO thwarted ICC progression through regulation of multiple oncogenes and suppressors. **(A)** Mining the differentially expressed genes in GSE33870, GSE32225 and GSE45001 using GEO2R. **(B)** 3D scatter of 35 common differentially expressed genes in the three data sets. **(C)** GO pathway analysis of 35 common differentially expressed genes in the three data sets. **(D)** The mRNA expression of candidate genes was detected by RT-PCR in shFTO TFK1 cells and corresponding control cells. **(E,F)** The mRNA stability of candidate genes was examined in FTO-overexpressing TFK1 cells, shFTO TFK1 cells, and corresponding control cells by RNA decay assay.

To unravel the molecular mechanisms meditating the biological functions of FTO, we analyzed microarray data obtained from the Gene Expression Omnibus (GEO) database (https://www.ncbi.nlm.nih.gov/geo/). In all, 1824 differentially expressed genes between *FTO*-depleted cells and scrambled cells were found by analyzing GSE33870 using GEO2R (*p* < 0.05, fold change>2, Figure 7A). The analysis of two ICC chips (GSE45001, 10 ICC samples and paired non-tumorous tissues; GSE32225, 149 ICC tissues and 6 non-tumorous tissues), (30, 31) revealed 3,246 differentially expressed genes in GSE45001 and 1580 differentially expressed genes in GSE32225 (*p* < 0.05, fold change>2, Figure 7A). Then, 35 differentially expressed genes were found to be overlapped from three databases (Figures 7A,B); these genes were determined to be involved in the integrin signaling pathway, inflammation mediated by chemokine and cytokine signaling pathways, the EGFR signaling pathway, angiogenesis and the pyrimidine metabolism pathway (Figure 7C). Further analysis of the expression status of 35 genes demonstrated that *FTO* knockdown induced the expression of *COL8A1, TEAD2*, and *CMTM4*, which were upregulated in ICC (Table 2) and that *FTO* knockdown inhibited the expression of *HAO2, NR5A2, CCL19, TCF21, APOA2, NTRK2, DPT FGA*, and *SCML4*, which were downregulated in ICC (Table 2). We further determined their roles in tumors. *TEAD2*, a transcriptional enhancer factor, contributes to EMT in breast cancer and pancreatic adenocarcinoma (PDAC) by directing its co-factor YAP1 to the nucleus (32, 33). *CMTM4* has been identified as a regulator of PD-L1 protein stability in tumor cells (34). The tumor suppressor gene *HAO2* inhibits hepatocellular carcinoma metastasis and predicts a favorable prognosis (35). Nuclear receptor *NR5A2* is associated with PDAC and transcriptionally regulates inflammatory gene expression (36). Chemokine *CCL19* inhibits colorectal cancer (CRC) angiogenesis in a CCR7-dependent manner (37). *TCF21* also acts as a tumor suppressors in multiple tumors, such as renal tumors, ovarian cancer, colorectal cancer, and lung cancer, among others (38–40). *SCML4* was reported to be downregulated in breast cancer stem cells (41). However, *APOA2, NTRK2*, and *FGA* are oncogenes in PDAC, glioblastoma, and gastric cancer (42–45). Thus, *FTO* may inhibit ICC progression by reducing oncogene expression (*TEAD2* and *CMTM4*) and inducing tumor suppressor expression (*HAO2, NR5A2, CCL19, TCF21, NTRK2*, and *SCML4*). Consistent with our analysis, in TFK1 cells, downregulation of FTO increased the mRNA levels of *TEAD2* (Figure 7D). Since FTO was demonstrated to regulate RNA stability, we tested the mRNA stability of *TEAD2* through RNA decay assay. The result showed that *TEAD2* mRNA stability was increased by inhibiting FTO in TFK1 cells (Figure 7E). Consistently, the mRNA stability of *TEAD2* was impaired by over-expressing FTO in TFK1 cells (Figure 7F).

**Table 2:** Genes related with ICC development based on FTO regulation.

**Gene symbol**	**GSE33870**	**GSE32225**	**GSE45001**	**Description**
COL8A1	6.589	2.515	7.516	Collagen alpha-1(VIII) chain
TEAD2	3.16	2.16	3.605	Transcriptional enhancer factor TEF-4
CMTM4	2.028	2.368	3.053	CKLF-like MARVEL transmembrane domain-containing 4
C7	0.49	0.397	0.038	Complement component C7
HAO2	0.444	0.374	0.005	Hydroxyacid oxidase 2
NR5A2	0.412	0.46	0.285	Nuclear receptor subfamily 5 group A member 2
CCL19	0.374	0.401	0.029	C-C motif chemokine 19
TCF21	0.339	0.419	0.349	Transcription factor 21
APOA2	0.274	0.293	0.002	Apolipoprotein A-II
NTRK2	0.203	0.454	0.158	BDNF/NT-3 growth factors receptor
DPT	0.162	0.465	0.027	Dermatopontin
FGA	0.136	0.461	0.031	Fibrinogen alpha chain
SCML4	0.066	0.328	0.358	Sex comb on midleg-like protein 4

## Discussion

The first m^6^A eraser, *FTO*, has long been considered an oncogene in tumors (18–20). However, in the present study, we showed that *FTO* functions as a tumor suppressor in ICC, which implies that FTO may be a context-dependent regulator in oncogenesis network. To explore the clinical relevance of the m^6^A modification in ICC, we screened the differentially expressed m^6^A readers, writers and erasers in para-HIBECS and ICC by IHC. *FTO* was found to be downregulated in ICC tissues and cell lines. In the clinical ICC samples, downregulated *FTO* was associated with the ICC biomarker CA19-9, which suggested that FTO negatively modulated the progression of ICC. Down-regulated *FTO* was also correlated with angiogenesis and MVD, which was demonstrated by CD34 expression. A Kaplan-Meier survival analysis showed that low *FTO* expression predicted poor prognosis in ICC. These clinical findings suggested that FTO played a suppressive role in ICC.

Previous study reported that *FTO* was highly expressed and functioned as an oncogene in certain subtypes of acute myeloid leukemia (AML) by targeting ASB2 and RARA through mRNA demethylation (18). However, their report also showed that *FTO* was downregulated in certain subtypes of AML, such as primary AML cases with MLL rearrangements/inv (16) or *t*_(8, 21)_ (18). Shun Zhou et al. also showed that *FTO* promoted chemo-radiotherapy resistance in cutaneous squamous cell carcinoma (CSCC) by targeting β-catenin through mRNA demethylation (20). These observations suggested that FTO might play dual roles in cancer. Furthermore, *FTO* and other m^6^A modification enzymes are also rarely mutated in cholangiocarcinoma, and their copy number changes are unknown. In the biological function analysis, forced expression of FTO markedly synergized cisplatin-induced apoptosis of ICC cells. Consistently, decreased endogenous expression of FTO reduced apoptosis of ICC cells. An anchorage-independent assay on soft agar demonstrated that *FTO* repressed the viability and growth of ICC cells. In addition, ectopic expression of *FTO* significantly hampered the mobility of ICC cells *in vitro*. Mechanistically, we turned to a GEO2R analysis using the public GEO microarray data. From the overlapped and differentially expressed genes from the three databases (GSE33870, 45001, 32225), we identified 35 differentially expressed genes. These genes broadly participate in multiple aspects of tumorigenesis, which suggests that FTO regulates the progression of ICC possibly through modulating the expression of these genes. Further research is needed to elucidate the mechanisms of FTO in the promotion of ICC. Recently, it has been reported that IDH mutations competitively inhibit RNA demethylation of FTO (21, 22). FTO is an α-ketoglutarate (α-KG)-dependent dioxygenase, which is competitively inhibited by R-2-hydroxyglutarate (R-2HG) and the structurally related metabolite D-2-hydorxyglutarate (D2-HG) (21, 22). In several studies, the IDH mutations promoted the accumulation of 2-HG in tumors, including ~20% of AMLs and ~15% of ICCs (5, 46). In ICC, the mutant IDH1/2 proteins convert alpha-ketoglutarate (αKG) to 2-hydroxyglutarate (2HG), which inhibits the activity of multiple αKG-dependent dioxygenases and results in alterations in cell differentiation and survival (47–49). These reports suggested that the mutated IDH1/2 promotes ICC by inhibiting the functions of FTO.

In summary, our study provides a novel insight into ICC development and suggests that down-regulation of FTO might establish a gene network that is in favor of ICC progression.

## Data Availability

All datasets generated for this study are included in the manuscript.

## Ethics Statement

All experimental protocols were approved by a Central South University institutional committee. Informed consent was obtained from all subjects. The study was reviewed and approved by the China national institutional animal care and use committee.

## Author Contributions

Z-XR, NL, Y-ZD, and L-QS designed experiments. Z-XR, ZL, and J-JH carried out experiments. Z-XR, L-YL, X-XR, JG, YM, and Y-DG analyzed experimental results and sequencing data. Y-DG and Y-MD assisted Z-XR with IHC CHIP assay. X-PZ, D-XZ, and NL gathered information of all patients. Z-XR wrote the manuscript.

### Conflict of Interest Statement

The authors declare that the research was conducted in the absence of any commercial or financial relationships that could be construed as a potential conflict of interest.
